# Mutant p53 as a guardian of the cancer cell

**DOI:** 10.1038/s41418-018-0246-9

**Published:** 2018-12-11

**Authors:** Fiamma Mantovani, Licio Collavin, Giannino Del Sal

**Affiliations:** 1 Laboratorio Nazionale CIB (LNCIB), AREA Science Park, Trieste, Italy; 2 Dipartimento di Scienze della Vita, Università degli Studi di Trieste, Trieste, Italy; 3 IFOM—the FIRC Institute of Molecular Oncology, Trieste, Italy

**Keywords:** Oncogenes, Ubiquitylation

## Abstract

Forty years of research have established that the p53 tumor suppressor provides a major barrier to neoplastic transformation and tumor progression by its unique ability to act as an extremely sensitive collector of stress inputs, and to coordinate a complex framework of diverse effector pathways and processes that protect cellular homeostasis and genome stability. Missense mutations in the *TP53* gene are extremely widespread in human cancers and give rise to mutant p53 proteins that lose tumor suppressive activities, and some of which exert trans-dominant repression over the wild-type counterpart. Cancer cells acquire selective advantages by retaining mutant forms of the protein, which radically subvert the nature of the p53 pathway by promoting invasion, metastasis and chemoresistance. In this review, we consider available evidence suggesting that mutant p53 proteins can favor cancer cell survival and tumor progression by acting as homeostatic factors that sense and protect cancer cells from transformation-related stress stimuli, including DNA lesions, oxidative and proteotoxic stress, metabolic inbalance, interaction with the tumor microenvironment, and the immune system. These activities of mutant p53 may explain cancer cell addiction to this particular oncogene, and their study may disclose tumor vulnerabilities and synthetic lethalities that could be exploited for hitting tumors bearing missense *TP53* mutations.

## Facts

- Mutant p53 oncoproteins are stabilized and activated in response to tumor-related stress stimuli.
- Mutant p53 orchestrates stress response mechanisms that facilitate tumor cell survival and adaptation to multiple intrinsic and extrinsic stress conditions (genotoxic, oxidative, and proteotoxic stress, hostile tumor microenvironment).
- The stress adaptive processes induced by mutant p53 trigger positive loops feeding its own accumulation in cancer cells.
- The crosstalk of mutant p53 and stress response mechanisms discloses therapeutic opportunities for treating tumors bearing missense *TP53* mutations.

## Open questions

- Which is the specific contribution of adaptive stress responses mediated by mutant p53 to the aggressiveness and mortality of different cancer types?
- Are there additional adaptive responses, yet to be identified, that can be supported by mutant p53 to influence cancer cell metabolism, proliferation, and interaction with the tumor microenvironment?
- To what extent does the crosstalk of mutant p53 and stress response mechanisms contribute to cancer cell addiction to mutant p53?
- Could pharmacological inhibition of specific stress response pathways be used to improve the efficacy of old and new drugs targeting mutant p53?

## Introduction

Tumors evolve through genetic and epigenetic changes that modify fundamental cellular programs of growth and proliferation, followed by selection of reprogrammed cells that best adapt to a variety of suboptimal or challenging conditions they encounter, either transiently or durably, during progression.

**Fig. 1:**
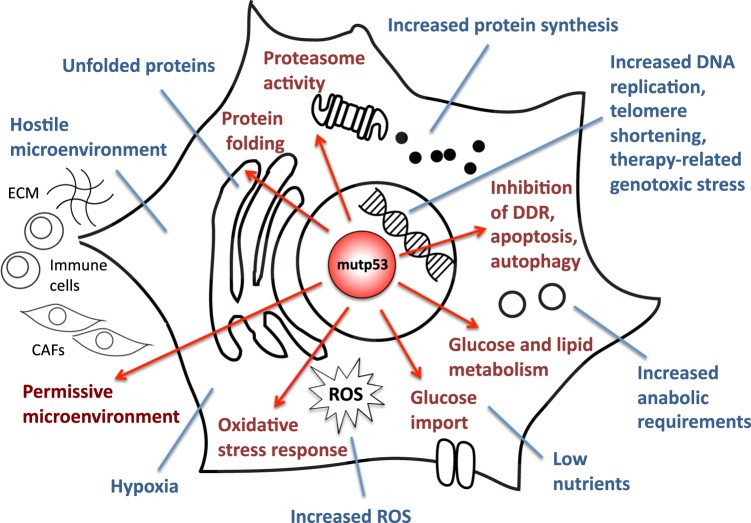
Mutant p53 promotes adaptive responses to cancer-related stress conditions to support tumor progression. Cancer cells in a growing tumor are exposed to multiple intrinsic and extrinsic stress conditions. Oncogenic p53 missense mutant forms (mutp53) can sense multiple stress inputs (blue), and act as homeostatic factors to induce adaptive mechanisms (red). Oxidative and proteotoxic stress: mutp53 has been shown to induce a pro-survival response to oxidative stress [45], to facilitate protein folding [69], and to increase proteasome activity [6, 66] in human cancer cell lines of breast, lung, and pancreatic origin. DNA lesions: mutp53 was shown to inhibit the DNA-damage response (DDR) in humanized mutp53 knock-in (HUPKI) mice [48, 49], to counteract autophagic cell death in breast cancer [25], and to inhibit therapy-induced apoptosis in head and neck cancer [55]. Altered metabolic requirements: mutp53 has been shown to sustain anabolic growth by enhancing glucose import and promoting the Warburg effect in mutp53 knock-in mice [34], and to modulate lipid metabolism in human breast cancer cell lines [42]. Hostile tumor microenvironment: mutp53 has been shown to modulate the extracellular milieu by promoting angiogenesis in breast cancer [73, 74], amplifying cancer-promoting inflammation in the colon of knock-in mice [79], and inducing a pro-invasive secretome in human lung tumors and derived cell lines [77]

The most frequently altered gene in human tumors is *TP53* [1], encoding the p53 protein. *TP53* mutations are associated with adverse prognosis in many sporadic cancers [1], moreover germline *TP53* mutations are causative of the Li Fraumeni syndrome, a rare familial cancer predisposition [2]. The primary outcome of *TP53* mutations is the loss-of-wild-type p53 functions, which represents a fundamental advantage during cancer development by depriving cells of intrinsic tumor suppressive responses, such as senescence and apoptosis. At variance with most other tumor suppressor genes however, the majority of *TP53* mutations are missense, producing single residue substitutions within the protein’s DNA-binding domain. p53 missense mutant proteins (hereafter referred to as “mutp53”) lose the ability to activate canonical p53 target genes, and some mutants exert trans-dominant repression over the wild-type counterpart. Beyond this, cancer cells appear to gain selective advantages by retaining only the mutant form of the p53 protein. This can be explained by the ability of different p53 mutants to reshape the tumor cell’s transcriptome and proteome, by virtue of newly established interactions with transcription regulators, enzymes and other cellular proteins [3, 4]. On this basis, specific missense p53 mutants have been reported to subvert crucial cellular pathways and to foster cancer cell proliferation and survival, promote invasion, migration, metastasis, and chemoresistance (reviewed in refs. [5, 6]). Whereas several mutp53 neomorphic phenotypes contributing to tumor aggressiveness have been described, our understanding of the mechanisms that determine cellular addiction to mutp53 expression for cancer maintenance and progression remains incomplete. Part of the tumor suppressive activities of wild-type p53 involves its capability to help the cell adapt to and survive mild stress conditions, including oxidative and metabolic stress [7]. Remarkably, mutp53 becomes stabilized and activated in response to tumor-related stress conditions, similar to the wild-type counterpart (see below). Alongside this notion, evidence is rising that mutp53 can provide cancer cells with the ability to cope with challenging conditions originated during tumorigenesis, including hyperproliferation-related DNA damage, oxidative and proteotoxic stress, nutrient fluctuations, physical constraints, stromal cues, and the anti-tumor immune response [8]. In this review, we propose and discuss the hypothesis that a major determinant of the cancer cell’s addiction to mutp53 derives from its ability to sense cell-intrinsic and extrinsic transformation-related stress conditions, and coordinate adaptive responses that support tumor progression (Fig. 1).

## Mutant p53 as a sensor of cancer-related cellular stress

**Fig. 2:**
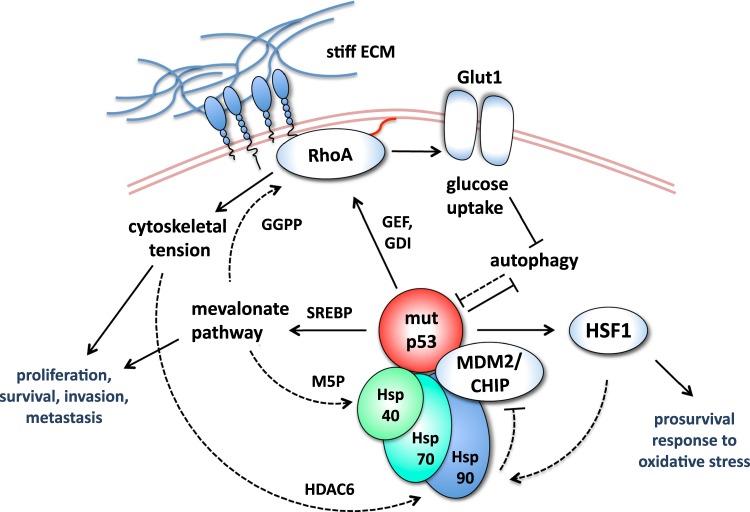
The stress adaptive processes induced by mutant p53 trigger positive loops feeding its own accumulation in cancer cells. In tumor cells, the Heat-shock protein (Hsp) chaperone machinery (comprising Hsp90, Hsp70, and Hsp40/DNAJA1) promotes mutp53 stabilization by inhibiting the ubiquitin ligases MDM2 and CHIP; mutant p53 can further enhance this mechanism by different means. In breast cancer cells, mutp53 was shown to induce the mevalonate pathway in concert with SREBP [42], thereby producing M5P that promotes interaction of mutp53 with Hsp40/DNAJA1 [44], as well as GGPP that stimulates RhoA activation and cancer cell mechano-responsiveness. In multiple tumor-derived cell lines, mutp53 was shown to promote RhoA activation also by inducing its positive regulators GEF-H1 [23] and RhoGDI [24]. Mechano-transduction activates the Hsp90 cofactor HDAC6, and this was shown to boost mutp53 stabilization in breast cancer cell lines and tumor xenografts [19]. Finally, in human and mouse breast cancer cell lines, mutp53 was shown to directly upregulate Hsp90/Hsp70 expression by stimulating HSF1 activity [14]. Besides the ubiquitin-proteasome pathway, mutp53 is degraded by autophagy-mediated proteolysis upon glucose deprivation [25]; in breast cancer models, mutp53 was shown to counteract the autophagic process [25]. Moreover, in various human cancer cell lines and in mutp53 knock-in mice, mutp53 was shown to increase intracellular glucose levels by stimulating RhoA-dependent membrane translocation of the Glut1 transporter [34]. M5P mevalonate-5-phosphate; GGPP geranylgeranyl-pyrophosphate

Similar to wild-type p53, mutp53 proteins are intrinsically unstable, due to proteasome-mediated degradation promoted by the E3 ubiquitin ligases MDM2 and CHIP [9]. However, in tumor tissues mutp53 proteins accumulate to elevated levels [10] and such stabilization is required for execution of their multifaceted oncogenic activities. Several studies clarified that in cancer cells the activity of MDM2 and CHIP towards mutp53 is specifically inhibited due to stable association of mutp53 with components of the Hsp90 chaperone machinery, a stress-induced system that supports cancer cell survival by counteracting protein misfolding and toxic aggregation (reviewed in ref. [11]). The system includes Hsp90, Hsp70 and other co-chaperones such as Hsp40/DNAJA1 (Fig. 2). Pharmacological blockade of this mutp53-stabilizing mechanism (Fig. 4) with inhibitors of Hsp90 and of its activator HDAC6 has been demonstrated to elicit mutp53 degradation in vitro and in vivo [9, 12, 13] and to increase tumor-free survival of mutp53 knock-in mice [13].

Similar to wild-type p53, whose accumulation and activation are triggered by transformation-related stimuli, an array of inputs originated within the altered tumor context conspire to induce mutp53 protein stabilization and oncogenic functions. Of note, some of the underlying mechanisms are supported by mutp53 itself, as will be detailed throughout this review.

During oncogenic transformation the Hsp90 system is frequently hyper-induced, due to activation of the master transcription factor heat-shock factor-1 (HSF1) in response to multiple stress conditions [14, 15]. Significantly, high levels of oxidative stress—a major inducer of HSF1—are frequently associated with tumor growth and were shown to cause mutp53 protein stabilization in vivo [16]. Moreover, mutp53 potentiates transcriptional induction of several heat-shock proteins by enhancing HSF1 stabilization and activation and participating into HSF1 transcriptional complexes at Hsp gene promoters [14], thus generating a feed-forward circuit that sustains mutp53 accumulation (Fig. 2).

The altered mechanical stimuli displayed by tumor tissues also contribute to cancer-specific accumulation of mutp53. Cancer-associated fibrosis generates a dense and stiff extracellular matrix (ECM) leading to integrin activation in focal adhesions and RhoA-induced remodeling of the actin cytoskeleton, which in turn favors tumor cell survival, proliferation and tumor progression [17]. It has been reported that mutp53 accumulation, often spatially heterogeneous within tumor tissues, is mainly observed at fibrotic regions [18], suggesting that the physical properties of the microenvironment may locally influence mutp53 stability. We have recently demonstrated that RhoA activation and actin-dependent mechano-transduction induced by elevated ECM stiffness promote activation of Hsp90 by the mechanosensitive HDAC6 deacetylase, thus leading to mutp53 stabilization [19, 20] (Fig. 2). RhoA is frequently over-expressed or hyper-activated in tumors [21]. Notably, mutp53 has been shown to promote RhoA activation [22] by inducing its positive regulators GEF-H1 [23] and RhoGDI [24], thereby sustaining aberrant tumor cell mechano-responsiveness as well as its own mechano-dependent stabilization (Fig. 2). An important implication of these findings is that preventing membrane recruitment and activation of RhoA by inhibiting its prenylation can block stroma-tumor mechanosignalling, providing a valuable approach to interfere with mutp53 stabilization in tumors [19, 20].

Nutrient limitations represent a major point of vulnerability of cancer cells. Interestingly, nutrient availability also appears to be relevant for mutp53 accumulation in tumors, as reduced glucose levels induce autophagy-dependent mutp53 proteolysis [25]. Of note, a dietary regimen of glucose restriction reduced mutp53 accumulation in mutp53(A135V) knock-in mice and reduced growth of mutp53-expressing tumor xenografts [25].

Multiple cancer-related stress stimuli trigger post-translational modifications of mutp53 to modulate its protein stability and interactions (reviewed in ref. [26]). For instance, constitutive activation of DNA damage checkpoint and consequent phosphorylation of Ser15 by ataxia-telangiectasia mutated (ATM) kinase drive stabilization of mutp53 by shifting the balance between its mono- and poly-ubiquitination [27]. This is consistent with the evidence that expression of Ras and Myc oncogenes as well as exposure to radiation- and chemotherapy-related genotoxic and oxidative stress induce accumulation of mutp53 in vivo [16]. Similarly, acetylation of C-terminal lysines has been shown to protect mutp53 from autophagy-mediated degradation [25]. Post-translational modifications of mutp53 also enhance its oncogenic activities: for instance, C-terminal phosphorylation by Polo-like kinase-2 (PLK2) was shown to boost mutp53 ability to promote breast cancer cells’ proliferation and chemoresistance [28]. We found that stress-induced phosphorylation at Ser/Thr-Pro sites, with subsequent Pin1-dependent isomerization, enhances the ability of mutp53 to neutralize p63 anti-metastatic activity and to induce a gene expression program associated with breast cancer aggressiveness and poor prognosis [29]. Pin1 has also been implicated in supporting gain-of-function of the p53-R249S mutant, frequently detected in human hepatocellular carcinoma (HCC) associated to dietary exposure to aflatoxin B1 and hepatitis B infection. In HBV-positive HCC, CDK4-dependent phosphorylation of the mutated Ser249-Pro250 preludes to Pin1-dependent modification, resulting in the interaction of p53-R249S with c-Myc to promote HCC growth [30].

## Mutant p53 rewires the cancer cell’s metabolism

Reprogramming of cell metabolism is a hallmark of cancer, required to sustain tumor cells’ biosynthetic needs for continuous growth and proliferation [31]. Moreover, cell metabolism is a central hub interconnecting the microenvironment, cell signaling and the epigenetic landscape, and is therefore crucial for cancer cells to cope with their changing environment [32]. Not surprisingly, cell metabolism is affected by multiple oncogenic conditions, including expression of mutp53.

One widespread metabolic adaptation of cancer cells is represented by increased glucose uptake accompanied by aerobic glycolysis (known as Warburg effect), which feeds tumor growth in hypoxic conditions and contributes to suppress immune surveillance through extracellular acidification [33]. Tumors hence display extremely high glucose requirements in face of nutrient scarcity and inadequate vascular supply (Fig. 1). Mutp53 has been reported to sustain glucose intake and hence the Warburg effect in tumor cells and knock-in mice, by the ability to induce membrane translocation of the glucose transporter GLUT1 via activation of the RhoA-ROCK axis [34] (Fig. 2). Remarkably, by promoting glucose intake in cancer cells, mutp53 also inhibits its own autophagy-dependent proteolysis, caused by glucose deprivation [25]. In addition, in response to energy stress mutp53 can induce aerobic glycolysis by directly inhibiting AMP-activated protein kinase (AMPK) [35].

Depending on the specific context, mutp53 can also promote oxidative phosphorylation, as shown in pre-neoplastic thymus and spleen of Li-Fraumeni (LF) mouse models and in muscles of LF patients [36]. Importantly, genetic or pharmacologic interference with mitochondrial respiration improved tumor-free survival in p53H172/+ mice [37], supporting the concept that mutp53-induced mitochondrial function enables malignant phenotypes. In contrast to the classic Warburg view, it has become evident that tumors display a significant degree of metabolic heterogeneity, and cancer cells can indeed activate either glycolytic or oxidative metabolism under different environmental conditions [8]. For instance, slowly proliferating tumor cells (e.g. circulating tumor cells) depend on mitochondrial function for energy metabolism and survival, rather than on “classic” glycolysis [38]. It is conceivable that mutp53 may endow cancer cells with metabolic plasticity, thus favoring their adaptation to metabolic stress and increasing their metastatic potential. Of note, a mutp53 R72 variant was recently shown to increase metastatic capability by stimulating mitochondrial function [39].

Many solid tumors undergo alterations of lipid metabolism, which contribute to cancer in multiple ways, e.g. by providing membrane lipids and supporting signaling pathways that promote proliferation and survival, EMT, cancer stem cells fate determination, and metastatic dissemination [40, 41]. Synergistic interaction of mutp53 with SREBPs, master regulators of fatty acids and cholesterol biosynthesis, leads to transcriptional induction of the mevalonate pathway (MVP) [42]. The MVP produces sterols and isoprenoids required for synthesis of membranes and lipid rafts, signal transduction and protein prenylation [41]. Several oncogenic outcomes derive from this activity of mutp53, including dismantling of normal mammary tissue architecture to facilitate tumor invasion [42] and promoting tumor cells’ aberrant mechano-responsiveness. In particular, MVP activity drives geranylgeranyl pyrophosphate (GGPP)-dependent RhoA activation (Fig. 2), which in turn promotes activation of the YAP/TAZ oncoproteins [43], as well as stabilization of mutp53 itself [19] (Fig. 2). In addition, induction of mevalonate-5-phosphate production along the MVP has been shown to cause mutp53 stabilization by stimulating its interaction with the Hsp40/DNAJ chaperone that inhibits mutp53 ubiquitination [44]. All these findings add on to the concept that, whereas modifying tumor cell’s adaptive processes, mutp53 induces positive regulatory loops feeding its own accumulation and oncogenic activities (Fig. 2).

## Mutant p53 enhances cancer cell survival under oxidative and genotoxic stress conditions

High levels of oxidative stress are frequently encountered during transformation as a consequence of genetic, metabolic and microenvironmental alterations, and preventing excess damage due to ROS accumulation is essential for cancer cytoprotection. The transcription factor NRF2 is central to control key components of endogenous antioxidant systems. Very recently, our group demonstrated that missense mutp53 interacts with NRF2 and contributes to selectively activating or repressing specific components of its transcriptional program, thereby promoting a pro-survival oxidative stress response that allows cancer cells to cope with high levels of intracellular ROS [45]. The mutp53-activated NRF2 target gene signature is associated with poor patient prognosis in breast cancer, and includes genes with pro-survival function, such as thioredoxin (TXN); in contrast, mutp53 represses other NRF2 targets including heme oxygenase 1 (HMOX1), which have been shown to display cytotoxic effects in cancer cells, although being cytoprotective in untransformed cells [46].

As a consequence of oxidative stress, DNA hyper-replication and telomere shortening, tumor cells endure persistent DNA lesions, leading to chronic activation of the DNA damage response (DDR), a tumor suppressive barrier that eliminates incipient cancer cells through either senescence or apoptosis [47]. Moreover, chemotherapy drugs represent major genotoxic stressors for tumor cells. Mutp53 interferes with DDR induction, by blocking the activation of the apical stress-sensor kinase ATM via disruption of the MRE11–RAD50–NSB complex [48, 49]. Loss of ATM function dampens homologous recombination (HR)-mediated DNA repair, rendering cells dependent on the activity of the enzyme poly(ADP ribose) polymerase 1 (PARP1). Interestingly, a proteomic analysis unveiled that mutp53 stimulates chromatin association and nuclear activity of PARP1, resulting in increased poly-ADP-ribosylated targets [50]. It is conceivable that, while blocking DDR activation on one hand, on the other hand mutp53 stimulates PARP function as a stress support mechanism allowing tumor cell survival in face of high levels of DNA damage. These activities likely underlie also mutp53-dependent adaptive responses promoting chemotherapy and radiotherapy resistance in tumor cells.

In addition, p53 mutants have been shown to boost HR-mediated DNA repair by enhancing topoisomerase 1 (Top1) function, although this activity has been reported to result in hyper-recombination and genomic instability [51]. It is also possible that mutp53 promotes chromatin recruitment of PARP, along with DNA replication factors PCNA and MCM4, to increase DNA replication efficiency [50]. Indeed, mutp53 has been recently shown to increase DNA replication origin firing and to stabilize replication forks, thus facilitating the proliferation of cells with genomic abnormalities. Consistently, mutp53 depletion leads to increased fork collapse in transformed cells [52]. Other adaptive mechanisms to proliferation-related stress may derive from DNA damage-induced association of mutp53 with DNA topoisomerase 2-binding protein 1 (TopBP1) [53], a scaffold protein that modulates DNA damage checkpoint, DNA replication and transcription [54]. Finally, mutp53 promotes cancer cell survival under tumor- and therapy-associated stress conditions by inhibiting the apoptotic and autophagic responses. mutp53 has been shown to promote resistance to chemotherapy in HNSC cancer by its ability to inhibit the p73 pro-apoptotic transcriptional program [55]; moreover, mutp53 can directly block caspase 9 activity [56], as well as caspase 9-mediated activation of mitochondrial caspase 3 [57]. p53 mutants have been demonstrated to suppress autophagy in tumor cells by various mechanisms. In particular, mutp53 directly inhibits the AMPK kinase, that under metabolic stress stimulates autophagy by phosphorylation of several transcription factors and metabolic enzymes, and stimulates the Akt/mTOR pathways in breast and pacreas cancer cells [58]. In addition to promoting autophagy resistance through cytoplasmic activities [59], inhibition of autophagy-related ATG12 gene by mutp53 has also been reported [58]. Of note, counteracting autophagy also protects mutp53 from proteolysis [60] (Fig. 2).

Adaptation to hypoxic conditions is a critical factor for tumor evolution. The cellular response to hypoxia is mainly regulated by the hypoxia inducible factor-1 (HIF-1). It has been reported that tumors bearing *TP53* mutations are generally characterized by higher HIF-1α levels [61], which can contribute to angiogenesis, chemoresistance, inhibition of apoptosis, and of autophagy. mutp53 appears to stimulate HIF-1α stabilization by inducing its dissociation from the ubiquitin-ligase MDM2 in hypoxic conditions [61]. This is consistent with the reported ability of mutp53 to inhibit the anti-metastatic p63 target gene Sharp1 [62], a factor that promotes ubiquitin-mediated degradation of HIF and blunts HIF-induced malignant cell behavior.

## Mutant p53 facilitates adaptation to proteotoxic stress

Proteotoxic stress in tumor cells arises as a consequence of enhanced protein synthesis and of gene mutations, including copy number alterations that change the stoichiometry of protein-complexes, and point mutations giving rise to aberrant peptides. All these events pose a high burden on the protein folding and degradation machineries of proliferating cancer cells.

**Fig. 3:**
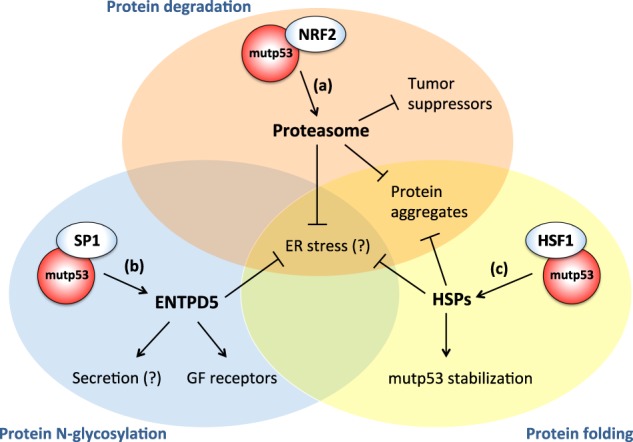
Mutant p53 facilitates adaptation to proteotoxic stress by multiple mechanisms. **a** In breast cancer cell lines, it was shown that mutp53 cooperates with NRF2 to induce expression of multiple proteasome subunits, accelerating turnover of tumor-suppressor proteins [66]. At the same time, increased proteasome activity contributes to alleviate stress caused by accumulation of misfolded proteins. **b** In pancreatic and breast cancer cell lines, it was shown that mutp53 cooperates with Sp1 to induce expression of ENTPD5, an enzyme involved in quality control of N-glycosylated secreted and membrane proteins, enhancing production of growth factors and growth-factor receptors [69]. At the same time, ENTPD5 may favor protein folding in the ER, and promote secretion. **c** Finally, in human and mouse breast cancer cell lines, it was shown that mutp53 cooperates with HSF1 to induce expression of various Hsp chaperones, contributing to alleviate proteotoxic stress, at the same time promoting mutp53 stabilization [14]

Accumulation of misfolded proteins in the ER activates the unfolded protein response (UPR), a conserved transcriptional program that helps resolve protein stress, but can also trigger apoptosis [63]. Cancer cells must therefore develop mechanisms that favor adaptation to protein stress, and limit apoptotic outcomes of pathways triggered by accumulation of unfolded proteins. Various experimental evidences suggests that mutp53 has a role in this process (Fig. 3).

The first such evidence is that mutp53 enhances activity of the proteasome. Earlier studies reported that mutp53 can upregulate expression of some proteasome subunits [50, 64], and of the proteasome activator REGγ [65]. More recently, in triple-negative breast cancer (TNBC) cell lines, we established that proteasome upregulation is in fact a highly conserved feature of oncogenic p53 mutants, correlating with enhanced protein degradation [66]. Mechanistically, this depends on the ability of mutp53 to interact with the transcription factor NRF2, and to selectively stimulate NRF2-dependent upregulation of proteasome subunit genes. The resulting enhanced proteasome activity in cancer cells bearing mutp53 increases their fitness and aggressiveness by accelerating the turnover of oncosuppressors such as CDK inhibitors or pro-apoptotic proteins, but also by enhancing resistance to proteotoxic stress. Of note, an augmented protein degradation capacity, associated to efficient elimination of misfolded nuclear and cytoplasmic proteins, was recently shown to be instrumental for transformation in various cell models. Strikingly, this capacity is mediated by NRF2-dependent upregulation of proteasome subunits and TRIM proteins [67].

The second evidence is that mutp53 upregulates Heat-shock proteins (Hsp). Li et al. [14] reported that mutp53 enhances stabilization and activation of heat-shock factor-1 (HSF1). In turn, mutp53 and HSF1 interact and bind together the promoters of several heat-shock proteins; thus, cells with mutp53 not only have increased HSF1 levels/activity, but also display an amplified transcriptional heat-shock response. Accordingly, cancer cell lines with mutp53 are more resistant to proteotoxic stress induced by heat shock, but also by proteasome inhibitors [14]. Moreover, HSF1-dependent accumulation of Hsps concurs to mutp53 stabilization in tumor cells [14], disclosing another feed-forward circuit instigated by mutp53. In addition to inducing chaperones, HSF1 coordinates a complex transcriptional response that affects cell proliferation, migration/invasion, and inhibition of apoptosis [68]. Thus, the interaction between mutp53 and HSF1 helps cancer cells to cope with proteotoxic stress on two fronts: by stimulating adaptation and by preventing apoptosis.

Finally, recent work associated mutp53 to the folding of glycoproteins in the ER [69]. This activity involves mutp53-dependent upregulation of ENTPD5, an enzyme of the calnexin/calreticulin chaperone system that guides folding of N-glycosylated proteins [70]. Notably, ENTPD5 depletion blocked invasion of mutp53-bearing cancer cells, while ENTPD5 overexpression rescued invasion of cancer cells depleted of endogenous mutp53, suggesting that ENTPD5 is a crucial mediator of mutp53 pro-metastatic gain-of function [69]. Experiments showed that the mutp53/ENTPD5 axis is required for maturation of N-glycosylated proteins and their transfer to Golgi compartment. Data suggest that maturation and processing of glycoproteins in the ER is fundamental for mutp53 oncogenic activity; the pro-tumorigenic effect of this process may be due to enhanced expression of membrane receptors, but also to enhanced secretion of extracellular mediators. This observation provides a first evidence that mutp53 may favor the folding of secreted and membrane proteins in the endoplasmic reticulum, possibly also contributing to alleviate the cytostatic effects of ER stress (Fig. 3).

## Mutant p53 facilitates establishment of a pro-oncogenic tumor microenvironment

Most tumors grow under a strong selective pressure from the surrounding environment. In particular during the invasion-metastasis process, cancer cells face stress conditions such as matrix detachment, interaction with altered stromal components, shear mechanical forces, and the presence of an anti-tumor immune response. The ability of cancer cells to actively shape a permissive microenvironment is thus crucial for cancer progression.

Increasing evidence indicates that mutp53 can remodel the tumor microenvironment, enhancing cancer cell adaptation to hostile extracellular conditions. First of all, mutp53 can stimulate tumor neo-angiogenesis. An old study showed that mutp53 overexpression induced VEGF in mouse fibroblasts [71]. Similarly, expression of mutp53 in bone marrow stromal cells increased production and secretion of VEGF, supporting the growth of leukemic cells [72]. Indeed, p53 mutation and VEGF levels are significantly correlated, at least in breast cancer [73]. In addition to VEGF, mutp53 can also increase cancer angiogenesis by upregulating ID4, a member of the ID family proteins. In turn, ID4 enhances post-transcriptionally the expression and secretion of pro-angiogenic cytokines IL8 and GRO1/CXCL1, stimulating tumor neo-vascularization [74].

Cancer cells secrete a variety of molecules that foster tumor growth and reseeding, and reshape the local microenvironment to facilitate invasion and metastatic dissemination. Analysis of mutp53-dependent tumor cell secretomes has suggested that mutp53-driven oncogenicity may act via regulating the expression of secreted proteins that function in either autocrine or paracrine signaling to induce migration and invasion of tumor cells [75]. Among these alpha-1 antitrypsin (A1AT) was identified as a critical effector of mutp53 in driving lung cancer invasion in vitro and in vivo, and correlated with adverse prognosis in mutp53-expressing lung adenocarcinoma patients [76].

Another feature of solid tumors is the presence of abundant inflammatory molecules secreted by cancer cells and by infiltrating immune cells. Inflammatory mediators can stimulate cell proliferation and motility, thus driving tumor aggressiveness; but factors such as TNF-α also convey powerful growth-restraining and pro-apoptotic signals. Thus, cancer cells must adapt to chronic inflammation by cutting the pro-apoptotic circuits, and amplifying the pro-survival and pro-migratory inputs of inflammatory signals. In this respect, mutp53 actively reshapes the profile of cytokines and chemokines secreted by cancer cells, contributing to establish a homeostatic microenvironment that eventually supports cancer cell growth and dissemination. For instance, mutp53 was reported to induce CXCL5, CXCL8, and CXCL12, correlating with increased cell migration and invasion, thus confirming that secretion of pro-angiogenic factors and chemokines is a gain-of-function of mutp53 [77].

The impact of mutp53 on the inflammatory tumor microenvironment is largely dependent on a functional interaction with the transcriptional regulator NF-κB. In fact, mutp53 was shown to promote p65 RelA nuclear translocation and amplify NF-κB transcriptional activity in cancer cells treated with TNFα [78–80]. Interestingly, mutp53 can influence TNF-induced NF-κB activation also by blocking the tumor suppressor protein DAB2IP, a cytoplasmic modulator that normally inhibits NF-κB and promotes TNF-induced activation of ASK1/JNK [81]. As a consequence, TNF stimulation of TNBC cells with mutp53 fails to induce apoptosis, but enhances cell migration and invasion [82].

The action of mutp53 on NF-κB may be particularly relevant in the context of the inflamed tumor microenvironment. Indeed, expression of mutp53 dramatically increased the incidence of invasive colon carcinoma in a mouse model of chronic colitis [79]. The activation of NF-κB sustained by mutp53 has two consequences: it amplifies the inflammatory response, by increased cytokine production, and it protects the cancer cell from cytotoxic effects of such microenvironment, by activating pro-survival pathways.

It appears that p53 mutation can also protect cancer cells from anti-tumor signals produced by other cell populations in the microenvironment. For instance, in vitro studies based on co-culture suggest that mutp53 protects cells from tumor-suppressive IFN-beta secreted by cancer associated fibroblasts (CAFs), enhancing survival, proliferation, and migration of lung carcinoma cell lines [83].

Finally, this concept might be extended even further, as we observed that a TNBC cell line expressing p53(R280K), when exposed to TNF, secretes chemokines that modulate recruitment of immune cells to the tumor [82]. More recently, it has been shown that mutp53-expressing cancers reprogram macrophages to a tumor supporting and anti-inflammatory state via exosomal secretion of miR-1246 [84]. These observations, suggesting that mutp53 could shape the tumor immune infiltrate, deserve further studies for their potential clinical implications.

## The homeostatic functions of mutp53 disclose therapeutic opportunities for cancer treatment

**Fig. 4:**
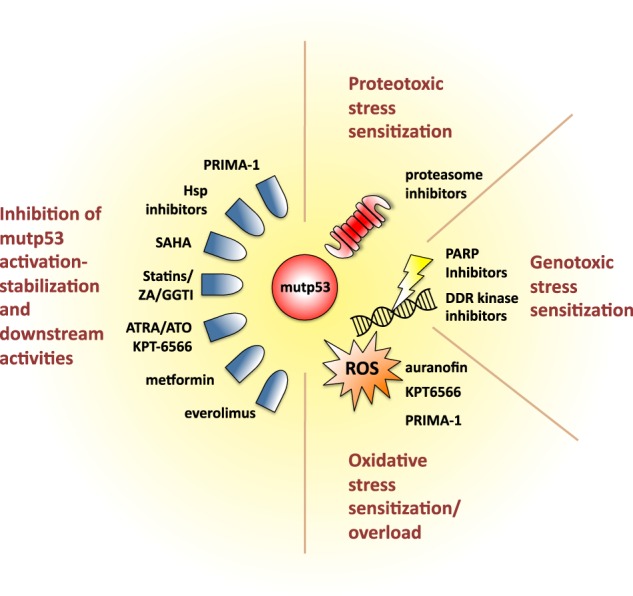
A schematic view of therapeutic opportunities targeting mutp53 and the homeostatic mechanisms it coordinates in cancer cells. Cancer cell addiction to mutp53-dependent stress support mechanisms can be exploited for therapeutic purposes by implementing pharmacologic strategies aimed at disrupting the balance of pro- and anti-survival signaling, in combination with molecules that directly target mutp53 and/or the mechanisms leading to its cancer-specific activation. PRIMA-1 is paradigmatic of small molecule compounds restoring mutp53 to its wild-type conformation and leading to its degradation. Destabilization of mutp53 by inhibition of the Heat-shock protein (Hsp) chaperone machinery can be obtained by different compounds, including inhibitors of Hsp90-Hsp40 and HDAC inhibitors (SAHA). Molecules inhibiting different steps of the Mevalonate-RhoA axis, including statins, Zoledronic Acid (ZA) and geranylgeranyl-transferase inhibitors (GGTI) can indirectly block Hsp90 activation and mutp53 stabilization, also blunting other oncogenic effects of this metabolic axis. Similarly, Pin1 inhibitors such as ATRA/ATO and KPT-6566 prevent mutp53 oncogenic activation in tumor cells. Metformin could block glucose-dependent mutp53 stabilization, and mTOR inibitors such as *Everolimus* curb tumor cell survival. Therapeutic approaches can be aimed to inhibit the stress support pathways sustained by mutp53 (sensitization), or to exacerbate stress conditions to overcome stress support pathways (stress overload). E.g. proteasome inhibitors blunt a major proteotoxic stress response pathway; inhibitors of the thioredoxin system (e.g. Auranofin) block antioxidant mechanisms; DDR kinase inhibitors and PARP inhibitors prevent responses to genotoxic stress; PRIMA-1 and the Pin1 inhibitor KPT-6566 increase ROS levels in cancer cells

There is ample experimental evidence showing that interference with mutp53 expression or activity by RNAi or pharmacological approaches leads to decreased cancer cell proliferation, survival and metastasis, and even causes tumor regression in vivo (reviewed in refs. [3, 85]). Given the uniquely high incidence of missense *TP53* mutations across many different tumor types, strategies aimed at blocking mutp53 would be expected to produce a huge impact on cancer treatment. However, anticancer therapies based on directly targeting mutp53 are still far from clinical practice: to date, the only compound that has successfully reached phase I/II clinical trials is the small molecule APR-246 (PRIMA-1MET), which reverts mutp53 to a wild type like conformation [86]. While doubts remain on whether mutp53 may in fact represent a tractable target, alternative therapeutic strategies may be envisaged to exploit tumor cells’ synthetic lethal interactions and dependencies on stress support mechanisms deployed by mutp53 (Fig. 4 and Table 1). These could become treatments of choice for tumors characterized by high *TP53* mutation rate and that still lack targeted therapeutic options, such as triple negative breast cancer and ovarian cancer.

**Table 1:** A survey of pharmacological approaches targeting mutant p53 and mutp53-dependent homeostatic circuits, detailing the experimental models or clinical settings in which they have been studied

Compound	Mechanism	Experimental model/clinical stage	Ref.
PRIMA-1MET	Binds to mutp53 restoring its wild-type conformation and activity and inducing its degradation.	Phase II clinical trials in ovarian cancer (NCT02098343; NCT03268382).	[86]
Hsp90 inhibitors, HDAC inhibitors (SAHA)	Elicit mutp53 destabilization by inhibiting the HDAC6-Hsp90 chaperone machinery to release MDM2/CHIP ubiquitin ligases.	Citotoxicity of the Hsp90 inhibitor 17AAG in combination with SAHA in mutp53 cancer cells is largely due to mutp53 destabilization.	[12]
		The Hsp90 inhibitor Ganetespib and combination of Hsp90 inhibitor 17-DMAG and SAHA restrained tumor growth in mutp53 R172H KI mice.	[13]
		AUY922 synergized with concurrent cisplatin radiotherapy in HNSCC with mutant *TP53* status.	[91]
Pin1 inhibitors (ATRA, ATO, KPT-6566)	Prevent activation of mutp53 downstream to phosphorylation-dependent prolyl-isomerization.	ATO/ATRA combination blunts Pin1 oncogenic activities and inhibits growth of TNBC cell lines and of patient-derived mouse xenografts.	[95, 96]
		KPT-6566 inhibits mutp53 oncogenic activities, decreases viability and metastatic capability of mutp53-expressing TNBC cells.	[98]
Mevalonate-RhoA pathway inhibitors (statins, ZA, GGTI)	(a) Elicit mutp53 destabilization by inhibiting the Hsp chaperone machinery; (b) Block MVP induction downstream to mutp53.	Statins and ZA decreased mutp53 accumulation in cancer cell lines and tumor xenografts, inhibiting tumor growth. Phase II clinical trials of pre-operative ZA (NCT02347163) and ZA/atorvastatin plus neoadjuvant chemotherapy (NCT03358017) in TNBC are ongoing.	[19, 43, 44]
Glucose-lowering treatments (e.g. Metformin)	(a) Interrupting glucose supply elicits mutp53 destabilization in tumor cells; (b) Metformin inhibits induction of OxPhos downstream to mutp53 blunting its oncogenic effects.	Glucose restriction inhibited mutp53 accumulation in mutp53(A135V) knock-in mice and reduced growth of mutp53-expressing tumor xenografts.	[25]
		Metformin treatment increased tumor-free survival in a Li Fraumeni mouse model.	[37]
Proteasome inhibitors (e.g. carfilzomib)	Inhibition of proteotoxic stress support pathways sustained by mutp53.	Carfilzomib synergized with PRIMA-1MET in reducing growth of breast cancer xenografts in a mutp53-dependent fashion.	[66]
PARP inhibitors, Chk1 inhibitors	Exploit dependency of mutp53-expressing tumor cells on PARP activity to counteract genotoxic stress.	mutp53 expression conferred sensitivity of cancer cell lines to cell death induced by Rucaparib.	[50]
		Chk1 inhibitor PF477736 inhibited growth of lung cancer xenografts in mice in a mutp53-dependent manner.	[52]
Inhibitors of antioxidant systems (e.g. Auranofin)	Inhibition of oxidative stress support pathways sustained by mutp53.	Auranofin synergized with PRIMA-1MET to selectively induce cell death in mutp53-expressing breast cancer cell lines; Auranofin effect in cancer is being evaluated in several clinical trials.	[45]
mTor inhibitors (e.g. Everolimus)	Inhibition of mTOR pro-survival pathway sustained by mutp53.	mutp53 expression conferred sensitivity of breast and pancreatic cancer cell lines to cell death induced by Everolimus.	[58]

For instance, the ability of mutp53 to inhibit autophagy and promote cell survival by stimulating the mTOR pathway sensitized mutp53-expressing breast and pancreas cancer cells to treatment with the mTOR inhibitor *Everolimus* [58]. Along this line, it has been recently shown that pharmacological activation of RAR-γ with a synthetic retinoid could circumvent resistance to p53 restoration in mutp53-bearing lymphomas [87].

Synthetic lethality of missense *TP53* mutations with blockade of DNA damage response and DNA repair pathways could be exploited to sensitize mutp53-expressing cells to chemotherapy. It has been shown that mutp53-bearing cancer cells are dependent on nuclear PARP activity for survival, and display increased sensitivity to treatment with the PARP inhibitor *Rucaparib* as compared to wild-type p53 cells [50]. Also, the PF477736 small molecule inhibitor of checkpoint kinase Chk1, which is induced by mutp53 to prevent collapse of DNA replication forks and lethal genome rearrangements, reduced the growth of lung cancer xenografts in mice in a mutp53-dependent manner [52].

The mutp53/NRF2 axis, enabling cancer cells to cope with high levels of intracellular ROS, also discloses implications for cancer treatment. In fact, loss-of-mutp53 impairs the activation of antioxidant systems, sensitizing cancer cells to cell death induced by excessive oxidative damage [45]. Interestingly, APR-246 PRIMA-1MET has been reported to increase ROS levels in cancer cells by targeting Thioredoxin reductase 1, independently of its action on mutp53 [88]. In fact, combined treatment with PRIMA-1MET and the FDA-approved compound *Auranofin* (inhibitor of the thioredoxin system) synergized in inducing cytotoxicity of breast cancer cells [45].

Similarly, mutp53 inhibition may sensitize tumors to proteotoxic stress. Tumor cells rely on the ubiquitin-proteasome pathway to reduce formation of toxic aggregates of misfolded proteins, and treatment with proteasome inhibitors has proven effective in malignancies, such as multiple myeloma and mantle cell lymphoma [89, 90]. As mentioned above, mutp53 protects cancer cells from proteotoxic stress by enhancing the expression of proteasome genes, which also confers resistance to proteasome inhibitors [66]. Treatment of mice with a combination of *Carfilzomib* and PRIMA-1MET reduced growth of breast cancer xenografts in a mutp53-dependent fashion [66]. Thus, clinical studies combining inhibition of mutp53 and proteasome activity may be hopefully envisioned in a near future. As mutp53 induces accumulation of Hsps, that in turn concur to mutp53 stabilization in tumor cells [14], treatment with Hsp inhibitors should induce mutp53 destabilization and concomitant sensitization of tumor cells to proteotoxic stress. Indeed, blocking Hsp90 activity with new generation inhibitors such as *Ganetespib* and the *Geldanamycin* derivative 17-DMAG, as well as the HDAC inhibitor *Vorinostat* (SAHA), restrained growth of mutp53-expressing tumors in vivo [13]. Supporting the clinical efficacy of this strategy, the Hsp90 inhibitor AUY922 was found to synergize with CCPT (concurrent cisplatin radiotherapy) in HNSCC cancers with mutant *TP53* [91].

Tumor associated metabolic reprogramming introduces metabolic liabilities that can be exploited for precision anticancer treatments [92]. For instance, the dependency of tumor cells on mutp53-induced glucose intake may be targeted to generate metabolic stress overload. Administration of glucose-lowering drugs, such as the anti-diabetic compound Metformin, could be effective also in that it interrupts the vicious circle whereby mutp53 stimulates glucose supply and sustains its own stabilization in tumor cells [25]. In fact, glucose restriction was shown to inhibit mutp53 accumulation in p53(A135V) knock-in mice, and to reduce growth of mutp53-expressing tumor xenografts [25], whereas Metformin treatment increased tumor-free survival in mutp53 R172H knock-in mice [37].

A similar reasoning can be applied to the MVP, whose induction both mediates tumorigenic activities of mutp53 and supports its accumulation in cancer cells [19, 44]. Indeed, blocking the MVP flux with statins [42, 44, 93], or preventing RhoA geranyl-geranylation by treatment with zoledronic acid (ZA) or inhibitors of protein geranylgeranyltransferase type I, such as GGTI-298 [19], effectively reduced mutp53 accumulation in cancer cells and dampened mutp53-dependent malignant phenotypes. Accordingly, administration of the clinically approved drugs *Atorvastatin* or ZA reduced growth of mutp53-expressing tumor xenografts in vivo [19, 44].

Finally, alternative pharmacological approaches may entail targeting druggable components of transduction cascades that convey tumor-related stress signals to mutp53. An example is blunting phosphorylation-dependent prolyl-isomerization of mutp53 catalyzed by Pin1, an enzyme frequently over-expressed in cancers [94]. For instance, HCC cells expressing mutp53(R249S) appeared to be highly sensitive to treatment with a CDK4 inhibitor that blunted Pin1-dependent activation of this particular p53 mutant [30]. While no specific Pin1 inhibitor has yet reached clinical trials, it has been discovered that All-trans Retinoic Acid (ATRA) and Arsenic Trioxide (ATO), used for treatment of acute promyelocytic leukemia, directly interact with the Pin1 active site, leading to inhibition and degradation of Pin1 in tumor cells, and to blockade of several Pin1-dependent cancer-driving pathways [95, 96]. ATO and ATRA synergyzed to inhibit growth of TNBC cells in vitro and of patient-derived orthotopic xenografts in mice [96]. However, ATRA has shown moderate efficacy against solid tumors in clinical trials [97], thus more effective Pin1 inhibitors are needed. We have recently isolated a novel Pin1 inhibitor named KPT-6566, able to covalently bind Pin1 and induce its degradation. This compound was shown to curb mutp53 GOF activities and decrease viability of mutp53-expressing cancer cell lines, as well as growth of lung metastasis of mutp53-expressing TNBC cells [98]. Remarkably, interaction of KPT-6566 with the Pin1 catalytic site releases a quinone-mimicking drug that generates reactive oxygen species, thus combining Pin1/mutp53 inhibition with oxidative stress overload in cancer cells.

## Concluding remarks

The stress phenotypes associated to tumorigenesis have been proposed to represent a common hallmark of cancer, and several oncogenic and non-oncogenic pathways were shown to generate adaptive responses that are essential for cancer cell survival [99]. Importantly, these pathways can be exploited to selectively kill cancer cells, through either stress sensitization or stress overload, with minor effects on normal cells. A wide array of neomorphic activities contributing to cancer progression has been attributed to missense p53 mutants: understanding the functions that generate tumor cell addiction in specific contexts is of primary interest to elaborate effective anticancer strategies. Here we have examined the abilities of mutp53 to support cancer cell survival under harsh conditions: these mirror basal functions of the wild-type counterpart, although being fulfilled through distinct mechanisms. We have hypothesized that these adaptive mechanisms may represent a major determinant of mutp53 oncogenic activity. Thus, mutp53-activated stress response pathways may be targeted to specifically sensitize, or overload, cancer cells bearing oncogenic p53 mutations to stress-inducing treatments. In other words, anticancer therapies based on drug combinations that either directly or indirectly hit mutp53-dependent homeostatic circuits can be expected to provide a number of exciting therapeutic possibilities (Table 1).

Clearly, the variety of *TP53* missense mutations produces distinct functional consequences, thus tumor vulnerabilities may differ based on the specific *TP53* mutation, as well as on the tumor type. Moreover, how the diversity of p53 isoforms reflects into oncogenic activities of mutp53 is presently a black box. Much study is still required to define these aspects, and we anticipate that multi-mutant, multi-omic approaches could provide a clearer perspective on the range of mutp53 cancer-protecting activities, and their prevalence in different mutp53 variants and in different tumor contexts, helping to identify “core” mutp53 activities as ideal therapeutic targets.
